# 植物精油成分气相色谱保留指数的全息定量构效关系

**DOI:** 10.3724/SP.J.1123.2023.07011

**Published:** 2024-04-08

**Authors:** Rui GUO, Long JIAO, Zubiao HU, Qingchen WANG, Hanbin ZHONG, Mingli JING

**Affiliations:** 1.西安石油大学化学化工学院, 陕西 西安 710065; 1. College of Chemistry and Chemical Engineering, Xi’an Shiyou University, Xi’an 710065, China; 2.中国石油川庆钻探工程有限公司长庆钻井总公司, 陕西 西安 710016; 2. Changqing General Drilling Company, CNPC Chuanqing Drilling Engineering Co. Ltd., Xi’an 710016, China; 3.西安石油大学电子工程学院, 陕西 西安 710065; 3. School of Electronic Engineering, Xi’an Shiyou University, Xi’an 710065, China

**Keywords:** 全息定量构效关系, 气相色谱保留指数, 植物精油, 分子贡献图, hologram quantitative structure-activity relationship (HQSAR), gas chromatographic retention index, plant essential oil, molecular contribution map

## Abstract

气相色谱保留指数(RI)是色谱分析中的重要参数,但通过实验获取RI值的过程较为繁琐,需要建立一种简便、高效、准确的模型来预测RI值。本文搜集了60种植物精油成分的RI实验值,构建了精油成分化合物的结构性质与RI值之间的全息定量构效关系(HQSAR)模型。当碎片大小(fragment size)、碎片特征(fragment distinction)和全息长度(hologram length)模型参数分别设置为“1~4”、“C, Ch”和199时,可以建立最优HQSAR模型。利用外部测试集验证和留一交叉验证对模型进行检验,经外部测试集验证的预测均方根误差(RMSEP)、预测决定系数( $Q_{F3}^{2}$ )、一致性相关系数(CCC)和平均相对误差(MRE)分别为40.45、0.984、0.968和2.20%;经留一交叉验证的交叉验证均方根误差(RMSECV)和MRE分别为72.56和4.17%。此外,HQSAR模型的分子贡献图表明,芳香族化合物的烷基链在连接了羟基基团后,其RI值会增大;脂肪族化合物中存在的长链烷基也会导致RI值增大。研究结果表明,所建立的HQSAR模型能够用于预测植物精油成分的RI值,并为其他精油成分RI值的预测提供可靠依据。

## Abstract (en)

The gas chromatography retention index (RI) is an important parameter for the identification of different types of compounds in the field of chromatographic analysis; however, the experimental collection of RI values is a extremely cumbersome process. Thus, there is an urgent need for the establishment of a simple, efficient, and accurate model for the prediction of the RI values of compounds. In this study, first, the experimental RI values for 60 plant essential oil constituents were obtained. Next, a model describing the hologram quantitative structure-activity relationship (HQSAR) between the structural properties of the essential oil constituents and their RI values was investigated and constructed. The optimal HQSAR model was established by setting the model parameters “fragment size”, “fragment distinction”, “hologram length” and “principal components” to “1-4”, “C, Ch”, “199”, and “4”, respectively. Finally, the predictive ability of the model was verified using external test set validation and leave-one-out cross-validation (LOO-CV). The experimental results were as follows, the root mean square error of prediction (RMSEP), predictive determination coefficient ( $Q_{F3}^{2}$ ), concordance correlation coefficient (CCC), and mean relative error (MRE) for external test set validation were 40.45, 0.984, 0.968, and 2.20%, respectively. Meanwhile, the root mean square error of cross validation (RMSECV) and MRE for LOO-CV were 72.56 and 4.17%, respectively. These findings demonstrate that the established HQSAR model has a good predictive ability and can accurately predict the RI values of plant essential oil constituents. In addition, the molecular contribution maps of the HQSAR model revealed that the RI values of aromatic compounds increase when hydroxyl groups are connected to their alkyl chains. Aliphatic compounds feature long chain alkyl groups, which can lead to an increase in RI values. The above phenomena highlight the promising application prospects of HQSAR for studying the RI values of plant essential oil constituents. Therefore, this study provides a reliable theoretical basis for predicting the RI values of other essential oil constituents.

植物精油又称挥发油,享有“液体黄金”的美誉,因具有抗氧化性、抑菌性等生物活性,成为医药、食品、化妆品、农业等领域的研究热点^[1,2]^。植物精油具有成分复杂、易挥发、热稳定性差等特点,色谱是研究这类化合物的重要手段^[3,4]^,国内外已有采用气相色谱法(GC)来分离精油成分的研究报道^[5,6]^。气相色谱保留指数(RI)是色谱定性的重要参数,能够反映物质在色谱固定相中的保留特性^[7,8]^。RI已被广泛应用于多种挥发性化合物的鉴别^[9,10]^,获取RI值的方法主要有两种,一种是通过实验计算所得,但利用该方法测定RI值的步骤繁琐,耗时长且成本高^[11,12]^;另一种则是通过对已知化合物的性质进行推测而得^[13]^。定量构效关系(quantitative structure-activity relationship, QSAR)^[14]^是色谱科学中的一个新兴研究领域,通过QSAR模型可以预测化合物的生物活性以及其他化学性质。目前,QSAR模型已被成功应用于预测精油成分的RI值^[15,16]^,但文献报道的模型都是基于2D-QSAR和3D-QSAR方法所开发的,通常需要计算大量的结构描述符或进行构象优化及分子叠合,建模过程复杂^[17]^。

全息定量构效关系(hologram quantitative structure-activity relationship, HQSAR)是建立在分子全息技术上,能快速、简便生成高质量QSAR模型的新方法^[18]^。与传统的2D-QSAR相比,HQSAR的结构描述符包含了更多的分子结构信息,预测能力更强;与3D-QSAR相比,HQSAR不需要进行构象优化和分子叠合,可以自动识别大批量化合物的结构性质,具有建模简单和计算速度快等优势。目前,HQSAR在生物学、医学、化学等众多领域都有着广泛的应用^[19,20]^,但关于植物精油成分RI值的HQSAR研究还未见报道。

本文在文献[21]的基础上,通过增加数据集的样本个数,建立了植物精油成分的结构性质与RI值之间的HQSAR模型,并根据所建立模型的分子贡献图来分析精油成分的结构性质对RI值的影响,验证了HQSAR模型在预测精油成分RI值中的实用性。

## 1 实验与方法

### 1.1 数据集

60种植物精油成分的RI实验值来自文献[21,22],详见表1。采用多次随机划分方法将60种化合物分为两组,第Ⅰ组(Group Ⅰ)为训练集,包含46种化合物;第Ⅱ组(Group Ⅱ)为测试集,包含14种化合物。数据集中的预测结果为多次随机划分的平均值。

**表 1:** 60种植物精油成分的RI实验值与预测值

No.	Compound	Experimental RI		Predicted RI		Relative error/%	
**Group** **Ⅰ**							
1	1-decanol (正癸醇)	1274.00		1279.45		0.43	
2	1-hexanol (正己醇)	868.00		909.38		4.77	
3	1-nonanol (正壬醇)	1173.00		1186.93		1.19	
4	alloaromadendrene oxid-(1) (香树烯环氧化物)	1625.00		1671.87		2.88	
5	α-bisabolol (α-红没药醇)	1443.00		1544.25		7.02	
6	σ-curcumene (σ-姜黄烯)	1510.00		1515.12		0.34	
7	(-)-σ-panasinsene (σ-人参烯)	1519.00		1582.85		4.20	
8	α-pinene (α-蒎烯)	932.00		1078.94		7.96	
9	α-selinene (α-瑟林烯)	1484.00		1422.11		-4.17	
10	β-cubebene (β-荜澄茄烯)	1376.00		1363.40		-0.92	
11	cis-lanceol (顺式-澳白檀醇)	1525.00		1587.69		4.11	
12	dodecanoic acid (月桂酸)	1564.00		1510.85		-3.40	
13	drimenin (金门苷)	1941.00		1881.46		-3.07	
14	drimenol (补身醇)	1770.00		1648.09		-6.89	
15	farnesene (法尼烯)	1453.00		1463.10		0.70	
16	hexanal (正己醛)	803.00		838.60		4.43	
17	humulene(律草烯)	1555.00		1678.88		7.96	
18	iso-caryophyllene (异丁香烯)	1737.00		1582.51		-8.90	
19	n-decanoic acid (正癸酸)	1373.00		1325.82		-3.43	
20	nerolidol (橙花叔醇)		1561.00		1549.45		-0.74
21	σ-cadinine (σ-檀香烯)		1496.00		1446.81		-3.29
22	tetradecanal (十四烷醛)		1614.00		1578.74		-2.18
23	trans-α-bergamotene (反式-α-佛手甘油烯)		1434.00		1459.46		1.78
24	trans-α-(Z)-bergamotol (反式-α-(Z)-香柠檬醇)		1606.00		1592.21		-0.86
25	trans-longipinocarveol (反式-长松香芹醇)		1634.00		1691.39		3.51
26	undecanal (十一醛)		1308.00		1301.19		-0.52
27	undecane (十一烷)		1101.00		1208.67		9.78
28	valencene (缬烯)		1489.00		1424.06		-4.36
29	xanthorrhizol (黄根酚)		1388.00		1467.80		5.75
30	1-octen-3-ol (1-辛烯-3-醇)		981.00		1002.94		2.24
31	linalool (芳樟醇)		1098.00		1227.08		11.76
32	borneol (冰片)		1167.00		1083.24		-7.18
33	nerol (橙花醇)		1233.00		1202.90		-2.44
34	thymol methyl ether (百里酚甲醚)		1239.00		1163.62		-6.08
35	neral (柠檬醛)		1245.00		1151.24		-7.53
36	geraniol (香叶醇)		1258.00		1202.90		-4.38
37	thymol (麝香草酚)		1298.00		1131.60		-12.82
38	methyl geranate (香叶酸甲酯)		1328.00		1268.47		-4.48
39	neryl acetate (乙酸橙花酯)		1368.00		1388.98		1.53
40	geranyl acetate (香叶乙酸酯)		1388.00		1388.98		0.07
41	trans-caryophyllene (反式-石竹烯)		1425.00		1582.51		11.05
42	germacrene D (大牛儿烯D)		1487.00		1508.33		1.43
43	β-bisabolene (β-红没药烯)		1515.00		1515.12		0.007
44	geranyl butanoate (丁酸叶醇酯)		1565.00		1557.97		-0.45
45	geranyl isovalerate (异戊酸香叶酯)		1606.00		1611.07		0.32
46	nonacosane (正二十九烷)		2895.00		2873.99		-0.73
**Group** **Ⅱ**							
47	1-dodecanol (十二醇)		1475.00		1464.49		-0.71
48	alloaromadendrene (香树烯)		1503.00		1542.73		2.64
49	α-caryophyllene (丁香烯)		1456.00		1468.88		0.88
50	β-carvophyllene oxide (β-石竹烯环氧化物)		1582.00		1604.11		1.40
51	decanal (癸醇)		1209.00		1208.67		-0.03
52	dodecanal (十二醇)		1413.00		1393.71		-1.37
53	(E)-caryophyllene ((E)-石竹烯)		1420.00		1421.13		0.08
54	farnesol (法尼醇)		1544.00		1609.63		4.25
55	isobornyl acetate (乙酸异龙脑酯)		1285.00		1239.31		-3.56
56	nonanal (壬醛)		1103.00		1116.15		0.92
57	β-himachalene (β-雪松烯)		1478.00		1568.51		6.12
58	nerol oxide (橙花醚)		1155.00		1223.10		5.90
59	geranial (柠檬醛)		1275.00		1251.24		-1.86
60	caryophyllene oxide (氧化石竹烯)		1587.00		1604.11		1.08

采用均方根相对误差(root mean square relative error, RMSRE)、交叉验证均方根误差(root mean square error of cross validation, RMSECV)、预测均方根误差(root mean square error of prediction, RMSEP)、预测决定系数(predictive determination coefficient, $Q_{F3}^{2}$ )、一致性相关系数(concordance correlation coefficient, CCC)等参数来评价所建立模型的预测能力^[23]^,具体计算过程如下:


(1) $$RE=\frac{x-\mu }{\mu }\times 100\%$$

(2) $$RMSRE=\sqrt{\frac{\sum {\left({RE}_{i}\right)}^{2}}{n}}\times 100\%$$

(3) $$\text{ RMSECV }=\sqrt{\frac{\sum_{i=1}^{n_{cv}}{\left(y_{i}-{\hat{y}}_{i}\right)}^{2}}{n_{cv}}}$$

(4) $$\text{ RMSEP }=\sqrt{\frac{\sum_{i=1}^{n_{EXT}}{\left(y_{i}-{\hat{y}}_{i}\right)}^{2}}{n_{EXT}}}$$

(5) $$CCC=\frac{2\sum_{i=1}^{n_{EXT}}\left(y_{i}-\bar{y}\right)\left({\hat{y}}_{i}-\bar{\hat{y}}\right)}{\sum_{i=1}^{n_{EXT}}{\left(y_{i}-\bar{y}\right)}^{2}+\sum_{i=1}^{n_{EXT}}{\left({\hat{y}}_{i}-\bar{\hat{y}}\right)}^{2}+n_{EXT}(\bar{y}-\bar{\hat{y}}{)}^{2}}$$

(6) $$Q_{F3}^{2}=1-\frac{\frac{\sum_{i=1}^{n_{ETT}}{\left({\hat{y}}_{i}-y_{i}\right)}^{2}}{n_{EXT}}}{\frac{\sum_{i=1}{\left(y_{i}-{\bar{y}}_{i}\right)}^{2}}{n_{TR}}}=1-\frac{\frac{\mathrm{PRESS}}{n_{EXT}}}{\frac{TSS}{n_{TR}}}$$

(7) $$q_{cv}^{2}=1-\frac{\sum {\left({\hat{y}}_{i}-y_{i}\right)}^{2}}{\sum {\left(y_{i}-\bar{y}\right)}^{2}}$$

式(1)~(7)中,*x*代表RI预测值,*μ*代表RI实验值,RE*_i_*代表第*i*个样本的预测相对误差,*y_i_*和 y^i 分别表示第*i*个样本的RI实验值和RI预测值, $\overset{-}{y}$ 和 $\overset{-}{\dot{y}}$ 分别代表RI实验值的平均值和RI预测值的平均值,*n*_cv_代表留一交叉验证的样本个数,*n*_EXT_和*n*_TR_分别代表外部测试集验证的样本数和训练集的样本数,PRESS代表预测误差的平方和,TSS代表全部样本的预测误差平方和,*n*代表样本总数, $q_{cv}^{2}$ 代表交叉验证相关系数值。在合理的QSAR模型中,CCC和 $Q_{F3}^{2}$ (F3为参数序号,在本文中无实际意义)一般应分别大于0.85和0.70^[23]^。

### 1.2 分子构建与结构优化

应用SYBYL-X 2.0软件(美国Tripos公司)来构建60种化合物的HQSAR模型,采用Tripos标准分子力场对化合物的分子结构进行优化,利用Gasteiger-Hückel方法计算原子电荷,其中能量收敛标准值设定为0.005 kcal/(mol·Å),迭代次数设定为1000次,其余参数选项均采用SYBYL程序的默认值。

### 1.3 HQSAR模型的建立

HQSAR的主要原理是通过建立化合物的分子亚结构片段(即分子全息)与性质之间的数学模型来预测未知化合物的性质^[18,24]^。建立HQSAR模型包括以下几个步骤:(1)将所研究化合物的分子结构拆分成M原子和N原子之间所有可能出现的分子片段(成环、线形、交叉、重叠等结构), M的最小取值从2开始,N的最大取值为12,且不能超过化合物分子中的原子数;(2)通过环丰度检验算法(cyclic redundancy check, CRC)对拆分完成的各个分子片段进行随机编码,所有编码数字均为正整数,且每个编码都对应一个具有全息长度(hologram length, HL)的整数排列,这些排列被称作分子全息;其中HL是在SYBYL程序设定的12个数值(53、59、61、71、83、97、151、199、257、307、353、401)中选择得到;(3)形成分子全息后,使用偏最小二乘法(partial least squares, PLS)构建化合物结构性质与分子全息之间的QSAR模型,最后以 $q_{cv}^{2}$ 值最高的模型作为HQSAR模型^[25]^。HQSAR模型的最优参数设置如下:碎片大小(fragment size)参数设定为“1~4”, 碎片特征(fragment distinction)参数设定为“C, Ch”,HL参数设定为199,最佳主成分数(principal components, PCs)为4。

## 2 结果与讨论

### 2.1 HQSAR模型优化

分子片段主要由fragment size和fragment distinction两个参数来确定^[26,27]^,其中fragment size代表分子片段中所包含的原子数目,实验时可以通过调整其数值大小来改变模型。fragment size参数中,1~3通常用来区分小型原子片段,可用于识别结构基团和原子类型;4~7代表中等大小的原子片段,可以对芳香环和脂肪环等官能团进行区分;8~10适用于大型原子片段。fragment distinction代表分子的片段特征信息,可选参数包括原子类型(atoms, A)、化学键类型(bonds, B)、连接类型(connections, C)、氢原子数(hydrogen atoms, H)、手性(chirality, Ch)、供体/受体原子(donor/acceptor atoms, DA)。

选择合适的fragment distinction和fragment size参数是构建高质量HQSAR模型的关键^[28]^,因此对这两个参数进行优化。以第Ⅰ组化合物为训练集建立HQSAR模型,首先设定fragment size参数为“4~7”,然后选择不同的fragment distinction参数组合进行建模,可以得到63个不同的模型,其中 $q_{cv}^{2}$ 最高的7个模型如表2所示。当fragment distinction参数选择“C, Ch”时可以得到最佳模型,此时 $q_{cv}^{2}$ 和决定系数(*r*^2^)分别为0.808和0.951。之后设定fragment distinction参数为“C, Ch”,改变fragment size参数进行建模,其中 $q_{cv}^{2}$ 最高的5个模型如表3所示。当fragment size参数设置为“1~4”时可以得到最佳模型,此时 $q_{cv}^{2}$ 和*r*^2^分别为0.897和0.948。通过上述研究可知,当fragment distinction和fragment size参数分别设置为“C, Ch”和“1~4”时,可以获得最佳HQSAR模型,此时HL和PCs分别为199和4。

**表 2:** 设置不同fragment distinction参数所得HQSAR模型

No.	Fragment distinction parameters		r^2^	PCs	HL
1	C, Ch	0.808	0.951	5	307
2	H, Ch	0.777	0.921	6	97
3	C, DA	0.764	0.957	5	307
4	A, C	0.761	0.930	5	307
5	A, B, C, H	0.584	0.943	6	307
6	A, B, C	0.582	0.929	5	307
7	B, C, H, Ch	0.550	0.940	6	151

$q_{cv}^{2}$ : cross validation coefficient; *r*^2^: coefficient of determination; PCs: principal components; HL: hologram length; C: connections; Ch: chirality; H: hydrogen atoms; DA: donor/acceptor atoms; A: atoms; B: bonds.

**表 3:** 设置不同fragment size参数所得HQSAR模型

No.	Fragment size parameter		r^2^	PCs	HL
1	1-4	0.897	0.948	4	199
2	2-5	0.884	0.968	5	199
3	3-6	0.861	0.973	6	307
4	4-7	0.808	0.951	5	307
5	5-8	0.560	0.942	6	307

### 2.2 HQSAR模型验证

HQSAR模型的分子贡献图可用于分析影响化合物性质的结构因素^[29,30]^。分子贡献图以不同颜色来表达基团对化合物结构性质的影响,黄色和绿色区域表示原子基团对分子性质产生正贡献,白色区域表示原子基团对分子性质不产生影响,红色和橙红色区域表示原子基团对分子性质产生负贡献。

采用留一交叉验证和外部测试集验证来检验HQSAR模型的稳健性和拟合能力。利用第Ⅰ组化合物完成留一交叉验证,并根据所建立的HQSAR模型对第Ⅰ组中46种化合物的RI值进行预测,结果见表1。46种化合物RI预测值的RMSRE为5.58, RMSECV为72.56,平均相对误差(mean relative error, MRE)为4.17%。RI预测值与实验值之间的线性关系为*y*=0.9478*x*+74.5281(*y*表示RI预测值,*x*表示RI实验值,相关系数(*R*)为0.9735), RI预测值与实验值之间的对比见图1。

**图 1:**
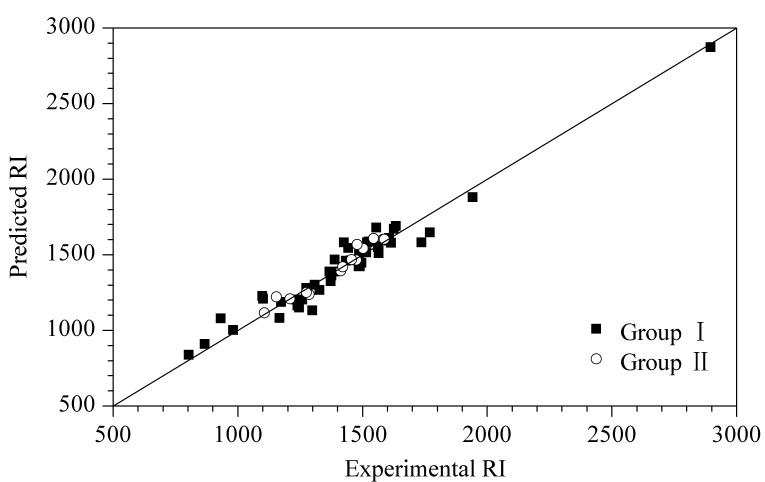
RI预测值与实验值之间的对比图

在外部测试集验证中,将第Ⅰ组化合物作为训练集建立HQSAR模型,并根据所建立模型来预测第Ⅱ组中14种化合物的RI值,结果见表1。14种化合物RI预测值的RMSRE为2.93, RMSEP为40.45, $Q_{F3}^{2}$ 为0.984, CCC为0.968, MRE为2.20%。RI预测值与实验值之间的线性关系为*y*=1.0552*x*-60.5292(*R*为0.9758), RI预测值与实验值之间的对比见图1。以上结果表明,本文所建立的HQSAR模型准确、可靠。

### 2.3 分子贡献图分析

HQSAR模型的分子贡献图如图2所示,11、14和24号化合物含有碳碳双键(C=C)和羟基(-OH)结构,与结构相似但不含有-OH基团的7、21和28号化合物相比,11、14和24号化合物的分子贡献图中绿色和黄色区域增多,RI值变大,说明芳香族化合物的烷基链在连接了-OH基团后,其RI值会增大。对比38和39号化合物发现,酯类化合物主链上连接的碳原子数增加后,分子贡献图中的绿色区域变多,RI值增大;26号化合物羰基(-C=O)位置上烷基链的长度小于22号化合物,分子贡献图中的绿色区域变少,RI值减小;对比1号和3号化合物可以发现,-OH连接的烷基链增长后,分子贡献图中的绿色区域变多,RI值增大。以上结果表明,脂肪族化合物中存在的长链烷基会使化合物的RI值增大。

**图 2:**
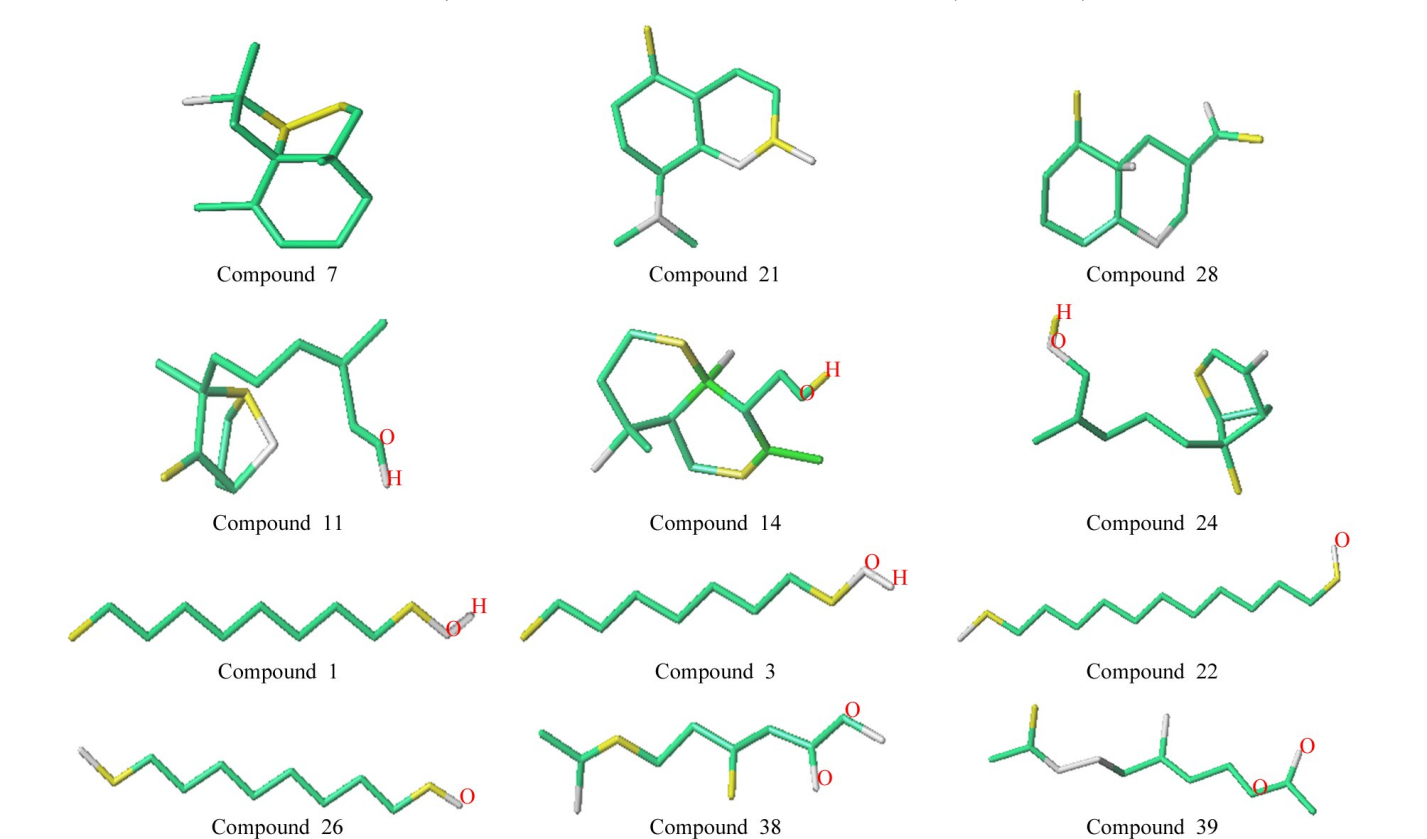
HQSAR模型的分子贡献图

### 2.4 方法比较

与2D-QSAR方法中的遗传算法(GA)^[31,32]^、人工神经网络(BPANN)^[33]^和多元线性回归(MLR)^[34,35]^相比,本文所建立的HQSAR模型增加了数据集的样本个数,建模方法简便,能够对影响精油成分RI值的结构因素进行全面分析。

## 3 结论

本研究构建了60种植物精油成分结构性质与RI值之间的HQSAR模型,该模型具有良好的稳定性和预测能力($q_{\mathrm{cv}}^{2}=0.897$,$r^{2}=0.948$),并能够很好地解释影响精油组分RI值差异的结构因素。实验结果表明,所建立的HQSAR模型可用于植物精油成分化合物RI值的预测,并为其他精油成分中未知化合物RI值的预测和计算机辅助分子设计相关研究的开展提供重要参考。
